# Clinical and Paraclinical Indicators of Motor System Impairment in Hereditary Spastic Paraplegia: A Pilot Study

**DOI:** 10.1371/journal.pone.0153283

**Published:** 2016-04-14

**Authors:** Andrea Martinuzzi, Domenico Montanaro, Marinela Vavla, Gabriella Paparella, Paolo Bonanni, Olimpia Musumeci, Erika Brighina, Hana Hlavata, Giuseppe Rossi, Gayane Aghakhanyan, Nicola Martino, Alessandra Baratto, Maria Grazia D’Angelo, Francesca Peruch, Marianna Fantin, Alessia Arnoldi, Andrea Citterio, Chiara Vantaggiato, Vincenzo Rizzo, Antonio Toscano, Nereo Bresolin, Maria Teresa Bassi

**Affiliations:** 1 IRCCS E. Medea, Polo Regionale di Conegliano, Conegliano (TV), Italy; 2 Fondazione CNR/Regione Toscana G. Monasterio, Unit of Neuroradiology, Pisa, Italy; 3 University of Messina, Department of Neurosciences, Messina, Italy; 4 IRCCS E. Medea, Neurorehabilitation Department, Bosisio Parini (LC), Italy; 5 Institute of Clinical Physiology, National Council of Research, Unit of Epidemiology and Biostatistics, Pisa, Italy; 6 ULSS 7-Pieve di Soligo, Department of Imaging, Conegliano (TV), Italy; 7 IRCCS E. Medea, Laboratory of Molecular Biology, Bosisio Parini (LC), Italy; 8 IRCCS Fondazione Policlinico, University of Milano, Department of Neuroscience, Milano, Italy; Oslo University Hospital, NORWAY

## Abstract

**Background:**

Hereditary spastic paraplegias (HSP) are a composite and genetically heterogeneous group of conditions mainly expressed by the impairment of the central motor system (“pure” forms). The involvement of other components of the central nervous system or of other systems is described in the “complicate” forms. The definition of an investigation protocol capable, by assembling clinical and paraclinical indicators to fully represent the extent of the motor system impairment, would help both the clinical handling of these conditions and contribute to our understanding of their pathogenesis.

**Methods:**

We applied a clinical and paraclinical protocol which included tools exploring motor and non motor functioning, neurophysiology and MRI to a composite cohort of 70 molecularly defined HSP patients aged 3 to 65, to define for each indicator its significance in detailing the presence and the severity of the pathology.

**Results:**

Clinically increased deep tendon reflexes and lower limb (LL) weakness are constant findings in all patients. The “complicated” forms are characterized by peripheral motor impairment, cognitive and cerebellar involvement. The Spastic Paraplegia Rating Scale efficiently reflects the severity of functional problems and correlates with disease duration. Neurophysiology consistently documents the impairment of the central motor pathway to the LLs. Nevertheless, the upper extremities and sensory system involvement is a frequent finding. MRI diffusion tensor imaging (DTI) highlighted a significant alteration of FA and MD. Combining the sampling of the various portion of the cortico-spinal tract (CST) DTI consistently discriminated patients from controls.

**Conclusion:**

We propose a graded clinical and paraclinical protocol for HSP phenotype definition, indicating for each tool the discriminative and descriptive capacity. Our protocol applied to 9 different forms of HSP showed that the functional impairment often extends beyond the CST. The novel DTI approach may add significant elements in disease recognition, staging and mapping.

## Introduction

Hereditary spastic paraplegia (HSP) is a clinical entity with a wide heterogeneity in molecular aetiology and a core homogeneous phenotype characterized by pyramidal tract disturbance of the lower limbs (LL) with spasticity and weakness, hyperactive deep tendon reflexes (DTR), extensor plantar responses, mild distal loss of vibration sense, inconstant urinary urgency [1]. Besides these core defining clinical signs, some forms called “complicated” are associated with other signs such as cerebellar ataxia, peripheral neuropathy, cognitive impairment, epilepsy, retinopathy, extrapyramidal manifestations, abnormalities on MRI, cataract and ichthyosis [2].

In spite of the apparently stereotypical clinical manifestation, a relevant variability is observed even among siblings or relatives in the same family [3]. In some cases this is explained by the association of more than one sequence variation of the same gene (Spastic Paraplegia Gene, SPG) [4–6], whereas in others subtle characteristics seem to differentiate one form to another [1, 3]: early onset associated with mutations of atlastin-1 (SPG3a) [7], *DDHD1* (SPG28) [8] and *REEP1* (SPG31) [9]; cognitive decline with mutations in spastin (SPG4) [10]; cerebellar signs such as ataxia with mutations of paraplegin (SPG7) [11]; axonal motor neuropathy and distal atrophy with mutation in seipin (SPG17) [12] and *KiF5A* (SPG10) [13, 14]; white matter (WM) MRI abnormalities with mutations of *CYP7B1* (SPG5) [15]; thin corpus callosum with a characteristic white matter alteration (“ears of lynx” sign) and intellectual disability in mutations of spatacsin (SPG11) [16]. Disease progression seems to differ between the various forms, as little or no progression is described in some (SPG3a), while a clear worsening even within few years is documented in others (SPG4) [17].

With over 72 loci and 55 verified genes [3, 18, 19] and given the absence of an effective treatment, there is a need to provide patients with an early clinical characterization and a more accurate prognostic information.

To this end, clinical and paraclinical indicators might help in defining and possibly predicting the clinical status and evolution of patients with HSP. However the relevance of each assessment procedure has not been systematically evaluated for the various HSP forms. Neurophysiological changes have been investigated in various cohorts of HSP patients and abnormalities in both the central and peripheral conduction parameters were associated with various HSP forms [20–22]. So far MRI studies have been used as exclusion criteria given the absence of specific findings on morphological examination (except for a thin corpus callosum characterizing in particular SPG11) [23].

Advanced neuroimaging MRI techniques have been recently more and more applied in various forms of motor neuron disease (MND). In particular, diffusion tensor imaging (DTI) indices variations (fractional anisotropy, FA, sensitive to microstructural changes associated with oriented structures in the tissue and mean diffusivity, MD, associated with the entities of obstacles to diffusion) have been studied in pure and complicated HSPs [24–27]. The ability of DTI to provide indications of the status of the cortico-spinal tract (CST), the “Achille’s heel” of the central nervous system [28], suggests that this technique may complement the clinical measures and provide a more accurate quantification of disease severity and progression.

In the last 10 years, we consecutively recruited and followed a large population of Italian patients with spastic paraplegia and we were able to molecularly characterize many of them. We herein present a full clinical and paraclinical characterization of this cohort of molecularly diagnosed HSP patients detailing how the various assessment tools may contribute to a more complete documentation and measure of the CST impairment.

## Materials and Methods

### Patients

Seventy patients with pure (43) and complicated (27) forms of molecularly defined HSP were recruited out of the 364 index cases followed in three locations in Italy (North-West, North-East, South). The demographic data and the molecular diagnosis of these subjects are presented in Table 1.

**Table 1:** Demographic data of the patients studied.

Genotype	n.	Gender (M)	Age at onset (yrs) mean ± SD (range)	Age at examination (yrs) mean ± SD (range)	Disease duration (yrs) mean ± SD (range)
***SPG3a***	7	2	0.64 ± 0.85 (0–2)	13.14 ± 13.02 (2–40)	12.5 ± 12.41 (2–38)
***SPG4***	32	22	34.96 ± 17.03 (1–64)	49.78 ± 17.15 (7–79)	14.81 ± 9.02 (3–33)
***SPG5***	7	1	24.14 ± 19.08 (8–54)	52.28 ± 13.53 (34–71)	28.14 ± 26.81 (1–60)
***SPG7***	4	2	45 ± 9.2 (34–54)	59.25 ± 13.86 (40–73)	14.25 ± 12.12 (6–32)
***SPG10***	3	2	22.33 ± 16.26 (4–35)	38.66 ± 9.02 (30–48)	16.33 ± 8.5 (10–26)
***SPG11***	11	4	14.36 ± 11.65 (3–46)	34.18 ± 8.6 (26–57)	19.81 ± 7.55 (10–31)
***SPG15***	3	0	14.66 ± 7.02 (8–22)	39.33 ± 8.96 (29–45)	24.66 ± 10.6 (15–36)
***SPG 31***	1	1	0.5	18	17.5
***SPG35***	2	1	38.5 ± 3.53 (36–41)	46 ± 1.41 (45–47)	7.5 ± 2.12 (6–9)
***Total***	70	35	25.97 ± 18.98(0–64)	42.97 ± 18.58 (2–79)	16.99 ± 12.5 (1–60)

**Abbreviations**: M, male; SD, standard deviation; SPG, Spastic Paraplegia Gene; yrs, years.

The study was approved by the Institutional Ethics Committee of “Eugenio Medea” Research Institute (# 63/09CE) and was conducted in accordance to the ethical standards of the Declaration of Helsinki (1964). All the adult participants and parents or legal tutors provided written informed consent prior to inclusion in the study. All the related documents were collected and stored from the main investigators of each center (AM, OM, GDA) according to the Institutional Ethic Committee guidance.

All patients followed the standardized clinical protocol, and most of them the paraclinical evaluation protocol described below. For all patients the age at onset and duration of symptoms (in years) were registered at the time of the first evaluation. All together there were 66 familial cases from 45 families and 8 sporadic cases.

### Molecular diagnosis

Molecular diagnosis was performed by direct sequencing (with a Big Dye Terminator Sequencing Kit -version 3.1 Applied Biosystem Foster City, CA, USA) of coding exons and splice site boundaries of all genes (SPG3a, SPG4, SPG5, SPG7, SPG10, SPG11, SPG15, SPG31and SPG35) in all patients. The mutations identified in all the patients are listed in the S1 Table and notes reported in the S1 File.

Four hundred healthy individuals from the Italian population were used as controls. SIFT, Provean, PoliPhen2, Mutation Taster, Mutpred, Pmut and Panther Software were used for pathogenicity prediction of the missense change. The missense change is not present in dbSNP, 1000genome databases and in the Exome Variant Server from NHLBI GO Exome Sequencing Project (http://evs.gs.washington.edu/EVS/).

### Evaluation protocol

The evaluation included clinical, neurophysiological and neuroimaging protocols. To assure homogeneity in the methodology, care was devoted to inter-center standardization of all procedures, except for the standard imaging which was completed with magnets of different strength in different locations.

### Clinical measures

Motor function and motility were explored with the spastic paraplegia rating scale (SPRS) [29], the modified Ashworth scale (MAS) [30], the 6 minutes walking test (6MWT) [31], LL DTR grading (0–4), LL muscle strength assessed with Medical Research Council (MRC) [32] megascore (MRC megascore for two flexors—hamstrings and gastrocnemius and two extensors—quadriceps and tibialis anterior bilaterally: range 0–40), distal muscle wasting (presence/absence).

Cognitive functioning was first assessed with the WAIS-R [33]. For a more refined assessment, 22 subjects showing borderline scores in some items were explored with the brief neuropsychological inventory 2 (ENB2) [34]. Independence in activities of daily living was explored with the functional independence measure (FIM) [35] as a widely diffused functional measure in disabling conditions. Eight patients (5 SPG3a and 3 SPG4) were children at the time of evaluation: for these subjects age appropriate assessment tools were used for the cognitive (WISC-R) [36] and functional evaluations [37]. The intellectual disability impairment and cognitive decline were diagnosed according to the definitions of the DSM-V [38].

### Neurophysiology

All collaborating patients in two of the participating centers (Conegliano and Messina) completed the neurophysiological protocol that included electromyography (EMG) and peripheral motor and sensory nerve conduction studies (NCS) of the four limbs; somatosensory evoked potentials (SSEP) of the LLs and motor evoked responses (MER) of the LL. SSEP and MER were extended to the upper limbs (UL) when the investigation in the lower limbs resulted abnormal.

Results for NCS and evoked potentials (EPs) were listed with the indication for NCS of either axonal or demyelinating pattern. The motor central conduction time of LL MER (mCCT) was also described and reported as informative parameters considering the normative values of each laboratory.

### Neuroimaging

Among the 70 patients studied, 58 underwent standard MRI studies for a qualitative morphologic evaluation, in the three centers. Standard MRI protocol included axial and coronal dual turbo- Fast Spin echo PD-T2 images and standard 2D T1-weighted spin-echo sequence on axial plane.

T1-weighted 3D isotropic images were obtained only in 36 of our patients: 14 with 3.0 T equipment (Bosisio Parini Centre, Philips Achieva 3T); 22 with a 1.5 T machine (Conegliano Centre, Philips Achieva 2.5 XR, Royal Philips Healthcare, Eindowen, NL)

**Fig 1:**
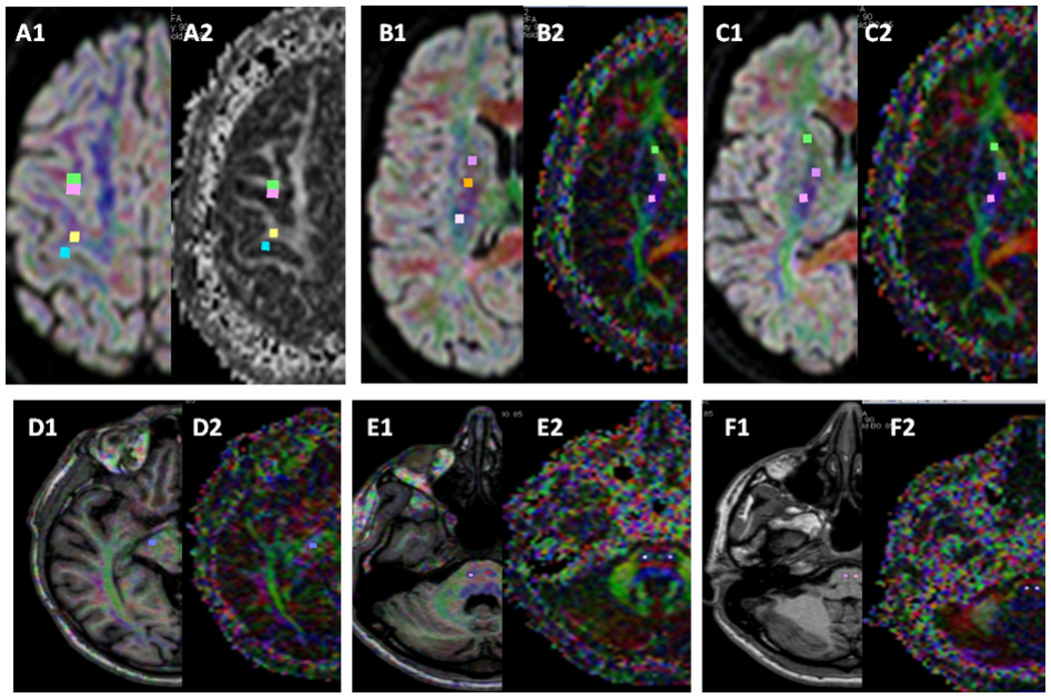
Examples of ROIs manually positioned along the cortico-spinal tracts (squared colored dots) for analysis of the diffusion values. Examples of displayed: on FA colored map superimposed to b = 0 image of the DTI acquisition (A1, B1, C1); on FA map (A2); on FA colored maps alone (B2, C2, D2, E2, F2). Anatomical ROIs positions: A1 and A2, vertex of sub-cortical pre-central and pre-motor WM and corresponding GM; B1 and B2, cranial portion of the internal capsule, close to centrum semiovale and corona radiata; C1 and C2, anterior and posterior limb, and genu of internal capsule; D1 and D2, cerebral peduncles; E1 and E2, pons; F1 and F2 bulbar pyramids. DTI acquisitions parameters: TR 10.000 ms, TE 69 ms; IR 2400 ms; EPI factor 55; acquisition matrix 104x102; voxel 2x2x2,03mm; b-value 0–1.000 s/mm; 102 contiguous slices. A 32 different gradient directions acquisition was applied and tensor reconstruction was obtained to create isotropic and anisotropic maps.

These last twenty-two HSP patients and twenty-two age-matched controls were selected exclusively in the Conegliano Centre and studied with the 1.5T machine in the Conegliano Center with DTI and H1-spectroscopy (MRS) acquisitions.DTI images were processed with a commercial workstation (Extended MR Workspace, ViewForum, Koninklijke, Philips Healthcare, NL, release 2.6.3.2) applying the *Fiber Track and Advanced Tools* software (Philips Healthcare, NL). The values for FA and MD indexes were obtained by manually defining individual regions of interest (ROIs) in consecutive slices (Fig 1), taking into account the rostro-caudal CST anatomical orientation, positioned at the following levels: vertex of sub-cortical pre-central and pre-motor WM and GM; anterior and posterior limb and genu of internal capsule close to centrum semiovale and corona radiata; anterior and posterior limb and genu of internal capsule; cerebral peduncles; pons; bulbar pyramids. Additional ROIs were placed on the genu and the splenium of the corpus callosum; in the WM and GM of the temporal, occipital, frontal and parietal lobes; in the WM of the cerebellar hemisphere and in middle cerebellar peduncles; in the central and anterior regions of both thalami; in both the amygdala and the hippocampal heads.

The ROIs were correctly placed in the various regions and in particular through the CST taking in account the *Fiber Track and Advanced Tools* resources allowing the contemporary visualization of the same anatomical region on b = 0 images of the DTI acquisition and the corresponding FA maps and color coded images.

In the same 22 patients and 22 normal age-matched controls that had DTI study, we performed a MRS study sampling in the pre-central regions. To define a peak table for N-Acetyl-Aspartate, Choline, Cr (Creatine-phosphocreatine complex) and myo-inositol, data were processed using *Spectro view software* (Philips Healthcare, NL), measuring peaks as ratio referred to the Cr, considered as normative inter-subjects units.

### Statistical analysis

For statistical analysis continuous data were expressed as means ± SD, while categorical data as percentage.

Multiple correspondence analysis (MCA) was performed to assess any possible clinical profile drawn from the correlation of additional signs and genotypes.

MCA is used to analyze a set of observations described by a set of nominal variables. It is a descriptive/exploratory technique designed to analyze multi-way tables containing some measure of correspondence between variables. MCA allows the analysis of the pattern of relationships among several categorical variables. MCA helps by transforming the dimensions of the data into a low-dimensional space where the largest amount of variability in the data points is captured in the first dimension, the next largest amount of variability in the second dimension and so on, maximizing the variability of data in a few dimensions. The results allow exploration of the structure of relationships between the categorical variables included in the analysis. One way to state the goal of a typical analysis is to represent variables in terms of distances between them in a low-dimensional space. The display of the variable points in the final coordinate system would provide an indication of the nature of the relationships between the variables.

The interpretation in MCA is often based upon proximities between points in a low-dimensional map (e.g., two or three dimensions). The proximity between levels of different nominal variables means that these levels tend to appear together in the observations.

Linear regression and multiple linear regressions were used to investigate the relationship between a continuous dependent variable and independent variables.

For DTI measurements, intra-observer and inter-observer reliability were determined using the intra-class correlation coefficient (ICC), adopting the classification derived by Hopkins criteria [39], described by Dini *et al*. [40]. These criteria defined ICC value from the lower (0.0–0.1), defined as “trivial”, to the highest (0.9–1), defined as “nearly perfect” agreement.

The comparison between the healthy group and HSP patients for DTI data referred to single ROI was performed by ANOVA (not adjusted p-value values). When heteroscedasticity was present in the data, the Welch ANOVA and the Wilcoxon test for unpaired data were used.

To discriminate the control group from the patients using FA and MD values of different anatomical tracts, a discriminant analysis was performed. The CST was anatomically a priori subdivided in contiguous portions as described in Fig 1. This parceling of the CST was extrapolated to evaluate the weight of single portion of the CST in order to discriminate HSP versus controls. This was done to identify the shortest portion of the CST that allows to correctly distinguish a HSP patient. FA and MD were considered as independent and conjunct factor. The percentage of correct classification, both global and specific for patients (sensitivity) and controls (specificity), and the area under the ROC curve (AUC) were computed. Pearson correlation was used to investigate the relationship among FA and MD, both in patients and controls.

A 2-sided p value <0.05 was considered to be statistically significant. JMP for Windows version 4.0 (SAS Institute Inc.) and SPSS version 21 were used to perform data analysis.

## Results

**Fig 2:**
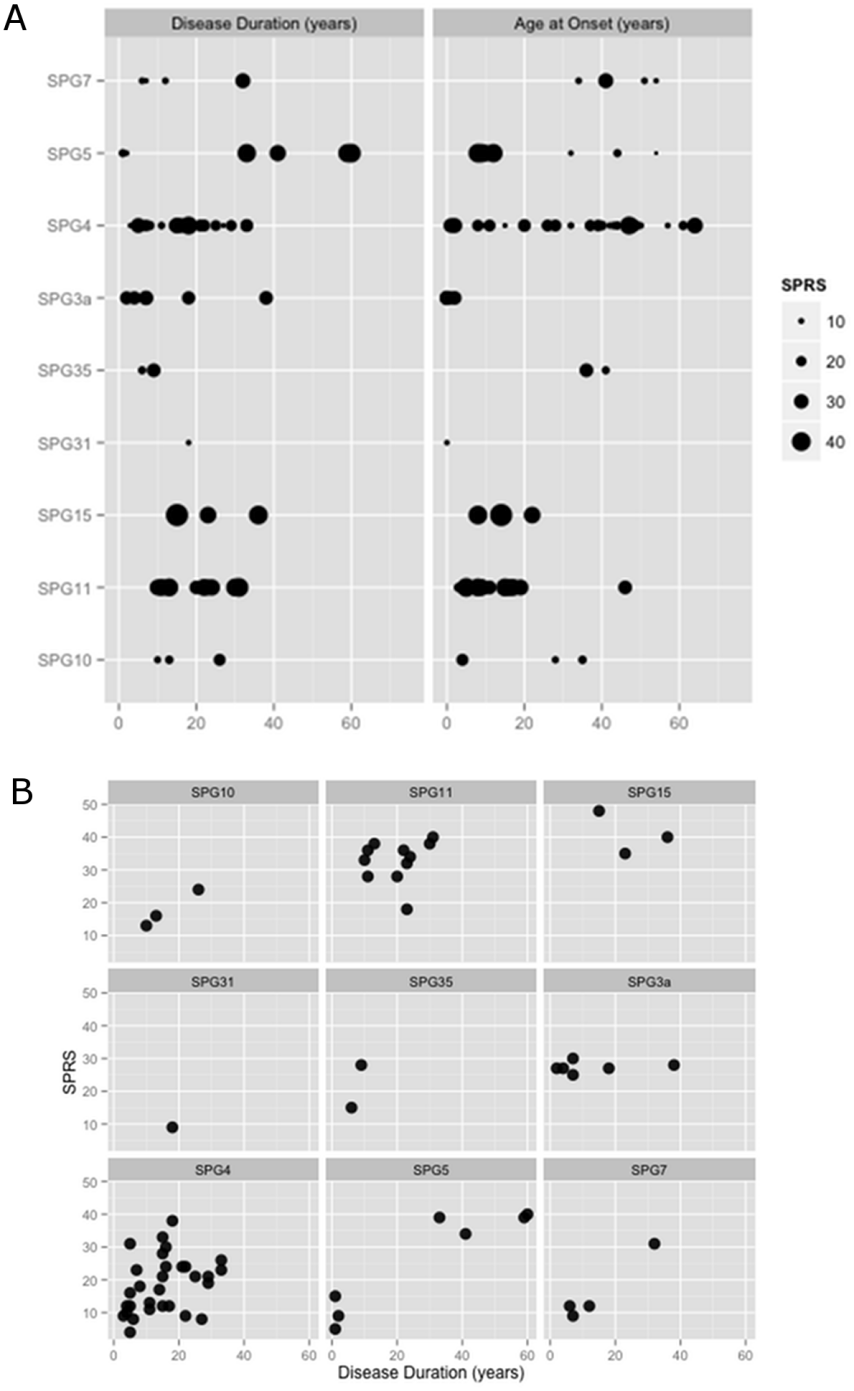
The disease duration and age at onset for the various SPG forms. (A) Dot-plot illustrates the disease duration and age at onset size-scaled by the magnitude of SPRS score for patients in different HSP forms. (B) Faceted scatter-plot with a matrix of panels. Each panel shows correlation between age of onset and disease duration and SPRS for different SPG forms. Abbreviations of Fig 2: SPRS, Spastic Paraplegia Rating Scale.

In this study males and females patients were equally represented (Table 1). The mean age at onset was 25.97 ± 18.98 years (0–64), but it widely varied among the various forms where SPG3a showed the earliest onset (< 1 year), SPG4, SPG35 and SPG7 the latest (35 to 45 years) (Fig 2). Duration of clinically relevant impairment reported at the time of evaluation was 16.99 ± 12.50 (range 1–60 years). The SPG35 patients showed the shortest duration (7.50 ± 2.12 years), while the longest was reported for SPG5 (28.14 ± 26.81 years) followed by SPG15 (24.66 ± 10.59 years) and SPG11 (19.81 ± 7.54 years). Considering the gap between age at onset and age of first visit (which for most cases corresponds to age at diagnosis) we observed a mean diagnostic delay of 15.22 years (range 1–41).

### Motor Clinical indicators

The LL MRC megascore indicated mild to moderate weakness (27.23 ± 12.30 out of 40) equally distributed among the various muscle groups (flexors, extensors, proximal, distal), in all cases (for additional information see the S2 Table). No difference was noted in the severity and distribution of weakness among different forms of SPGs, apart from patients with SPG3a, where weakness was more severe.

The LL MAS score was increased (2.04 ± 0.98) in all patients but 15 (four SPG4, three SPG7, two SPG11 and two SPG35, one for each of SPG3a, SPG5, SPG10 and SPG31), where it was 1 to 0.

The LL DTRs were all above 3 in every symptomatic patient, including those not showing increased MAS score. Muscle wasting was the most common additional sign, observed mainly distally in over a third of patients (25) and in all forms but SPG10 and SPG31:patients with SPG15 (all), SPG11 (9/11), SPG5 (4/7) and SPG7 (3/4) were the most frequently affected. Muscle wasting was, most of the times, associated with a motor axonal neuropathy (22.8% of all patients and 64% of those with muscle wasting showed neuropathy).

The type, frequency and distribution of the other neurological and non-neurological signs are summarized in Table 2. Cerebellar signs (dysarthria, dysphagia, ataxia) were the next most common additional findings, followed by cognitive impairment. Cognitive impairment was considered as a cognitive decline not present since birth or very early life while IDD is defined as a condition with onset during the developmental period including both intellectual and adaptive functioning deficits [38]. In our cohort we found IQ was <1 SD below normal average in 12 subjects, mostly SPG11 (8) and SPG15 (2). Subtle difficulties were spotted in selected areas of routine cognitive testing (trail making test B, Rey’s figure) in 22 patients (2 SPG3a, 14 SPG4, 4 SPG5, 1 SPG11 and 1 SPG31) who showed a IQ within the normal range. These patients were further investigated with the ENB2 battery revealing below threshold functioning (<66) in one patient with SPG3a, five SPG4 and one SPG11. Optic atrophy as judged by ophtalmoscopic evaluation was seen only in SPG11 and SPG5 and epileptic seizures in a single SPG15 case. Among non-neurological signs only early cataract was reported in one SPG5 and one SPG35 case.

**Table 2:** Additional clinical signs in the various SPG forms.

Genotype	n.	Ataxia	Dysarthria	Dysphagia	IDD (*)	Cognitive impairment (**)	Epilepsy	Deafness	Optic atrophy (***)	Early cataract
***SPG3a***	7	-	-	-	-	1 (14.3)	-	-	-	-
***SPG4 (5)***	32	2(6.3)	1 (3.1)	-	1 (3.1)	5 (15.6)	-	-	-	-
***SPG5***	7	2 (28.6)	-	3 (42.9)	-	-	-	2 (28.6)	1 (14.3)	1 (14.3)
***SPG7 (6)***	4	3 (75)	-	-	-	-	-	-	-	-
***SPG10***	3	-	-	-	1 (33.3)	1 (33.3)	-	-	-	-
***SPG11***	11	9 (81.8)	6 (54.5)	6 (54.5)	8 (72.7)	2 (18.2)	-	-	2 (18.2)	-
***SPG15***	3	3 (100)	2 (66.7)	1 (33.3)	2 (66.7	-	1 (33.3)	-	-	-
***SPG31***	1	-	-	-	-	-	-	-	-	-
***SPG35***	2	2 (100)	2 (100)	-	-	2 (100)	-	-	-	1 (50)
***Total***	70	21 (30)	11 (15.7)	10 (14.3)	12 (17.1)	11 (15.7)	1 (1.4)	2 (2.9)	3 (4.3)	2 (2.9)

***Note***: The numbers in parentheses are frequencies (%).

(*) IDD, Intellectual Developmental Disorder [38];

(***) Includes one case with impaired VEP;

(**) Mild cognitive impairment with ENB-2 < 66 [38]; SPG, Spastic, Paraplegia Gene.

**Fig 3:**
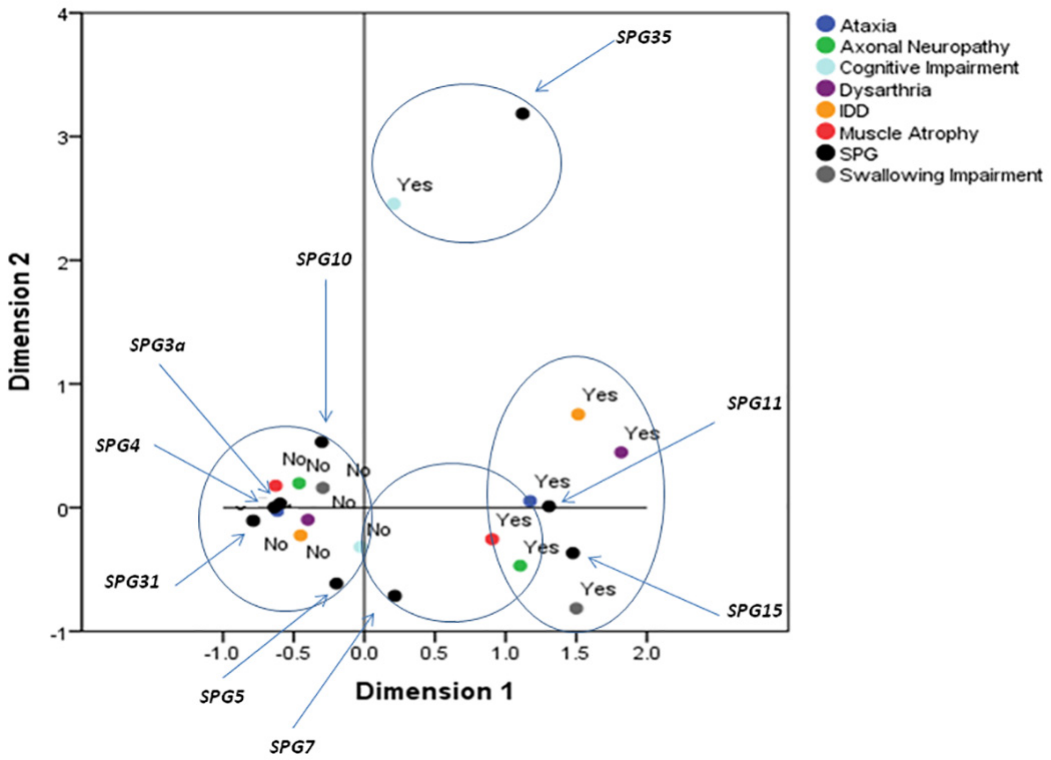
MCA analysis of additional signs reported in HSP patients. In the space defined by the first two dimensions emerge three different profiles: bottom left (SPG3a, SPG4, SPG5, SPG10 and SPG31) absence of all the reported variables; bottom right (SPG11 and SPG15) presence of muscle atrophy, axonal neuropathy, ataxia, dysarthria, dysphagia and cognitive impairment; cloud between the first and second group (SPG7) presence of muscle atrophy, axonal neuropathy and ataxia. Top right (SPG35) cannot be considered significant as including only two individual with cognitive impairment. The genotypes are represented by black dots, arrows link to the specific SPG type. IDD: intellectual disability impairment. Abbreviations of Fig 3: IDD, Intellectual Developmental Disorder; SPG, Spastic Paraplegia Gene.

When the additional signs muscle wasting, peripheral neuropathy, ataxia, dysarthria, dysphagia and cognitive impairment, were considered as binary variables for MCA (Fig 3) 4 clouds consisting in 3 groups of profiles emerged in the space defined by the first two dimensions. The first group (see cloud on bottom left in Fig 3) includes the grouping of the following genotypes SPG3a, SPG4, SPG5, SPG10 and SPG31 which appears to be characterized by the absence of all the reported variables. The second group (see cloud on the bottom right) includes the SPG11 and SPG15 genotypes and appears to be characterized by the presence of all the considered variables. The third and last group (see cloud between the first and second group) includes SPG7 and appears to be characterized by the presence of muscle atrophy, neuropathy and ataxia. A fourth cloud emerges in top right, represented by the SPG35 and mainly characterized by the cognitive impairment; however, since the cloud includes only two individuals, it has not been considered.

### Functional composite measures

The 6MWT was applicable to 40 ambulant patients (for additional information see S2 Table). In all tested patients the distance covered was significantly decreased (mean 220.82 ± 104.37 m) compared to the predicted distance for matched healthy controls (593 ±57 m) [41]. The most severe limitation was seen in ambulant SPG5 patients (64.80 m), the mildest in SPG4 (412.20 m). However, a wide variation of distance covered was observed within subjects belonging to the same SPG form (e.g. distances covered by SPG4 patients were between 66.00 to 412.20 m, and distances covered by SPG5 patients were between 64.80 to 375.00 m). Remarkable variation was seen even among members of the same family: two siblings with a SPG5 diagnosis walked the longest (360.00 m) and the shortest (64.80 m) distance among all SPG5 patients.

Mean SPRS score indicated moderate involvement (mean score 23.27 ± 10.7, range 4–48), with the greatest variability seen in SPG5 and the smallest in SPG3a. There was a significant correlation between SPRS and temporal variables such as age at onset and disease duration (model R2 = 0.327, p<0.0001, n = 69; SC ß = -0.276, p = 0.014 for age at onset; SC ß = 0.408, p<0.0001 for the reported disease duration).

The total FIM score was severely decreased only in SPG11 (55/126), while it was between 80 and 93/126 in SPG7, SPG10 and one SPG4, and above 104/126 in all the others. When a multiple linear regression with FIM as dependent variable was performed, FIM did not show any significant correlation with the temporal predictors such as age at onset and disease duration. At univariate analysis by linear regression FIM showed a significant correlation with the SPRS as a predictor (model R^2^ = 0.590, n = 36, SC *ß* = -0.768, p<0.0001). Finally, when a multiple linear regression was applied (model R^2^ = 0.504, *p* = 0.001, n = 23), FIM showed a significant correlation with: SPRS (SC *ß* = -0.420, *p* = 0.028) and ENB2 (SC *ß* = 0.412, *p* = 0.030).

### Neurophysiology

EMG and NCS were performed on 49 patients. Distal axonal motor neuropathy and neurogenic changes were detected in 23 subjects: most of the SPG7, SPG11, SPG15 and SPG35 patients, the eldest three SPG3a patients, a minority of SPG4 (5 out of 23) and one SPG5 subject. There was a good overlap between presence of neuropathy and muscle wasting and hypotrophy. he LL SSEPs were performed in 44 patients; the results showed abnormalities (increased latency of N22 and P40) in 30 of them (68%) belonging to all SPG types except SPG10, SPG31 and SPG35. The ULs SSEP were examined in the subjects showing altered LL parameters, and were found abnormal in 21: a third of SPG4 tested patients (9/22) and in most of the SPG5, SPG7 and SPG15 patients (90%). Normal UL SSEP were found in all SPG3a and SPG11 patients.

The LL MERs were abnormal with responses either absent (three cases) or significantly delayed (mCCT increased with a z-score of 19.85 ± 15.71) in all but one SPG4 patient with a very mild phenotype. The ULs MERs were tested in 31 subjects and found abnormal in 14 (45%): 6 SPG4, 3 SPG11, 2 SPG5 and in one patient of these 3 forms: SPG7, SPG15 and SPG35 although not paralleled by any clinical involvement.

### Neuroimaging

For routine clinical purposes, visual inspection and qualitative assessment of the MRI images revealed on standard morphological MRI studies (Table 3) normal aspects in slightly more than a third of all tested patients; in two cases of SPG5 alterations in deep supratentorial WM were found with demyelinating aspects; diffuse or focal brain atrophy were distributed for all SPG types; one SPG4 case had an acoustic Schwannoma. The “ear of the lynx” sign described as typical for SPG11 [42] was indeed detected in the majority of SPG11 patients but also in one SPG7 patient.

**Table 3:** Findings on the standard MRI examinations.

Genotype	n.	WM alterations	Cortical atrophy	Corpus callosum abnormalities	Ears of the lynx sign
**SPG3a**	5	0	0	1	0
SPG4 (*)	27	5	12	3	0
**SPG5**	6	4	3	0	0
**SPG7**	4	1	4	1	1
**SPG10**	2	0	0	0	0
**SPG11**	10	8	4	7	8
**SPG15**	1	1	0	1	0
**SPG31**	1	0	0	0	0
**SPG35**	2	0	2	2	0
**Total**	58	19	25	15	9

**Abbreviations**: SPG, spastic paraplegia gene; WM, white matter.

(*) One schwannoma of VII-VIII of cranial nerves.

The results of H-MRS samplings were inconclusive and are presented in the S1 File.

DTI data were tested for ROI reproducibility validation. The magnitude of intra- and inter-observer correlation, according to Hopkins criteria, varied from “very large” to “nearly perfect”. The ICC variability was: intra-observer 0.95 and inter-observer 0.99 for FA, and 0.99 and 0.85 for MD.

**Fig 4:**
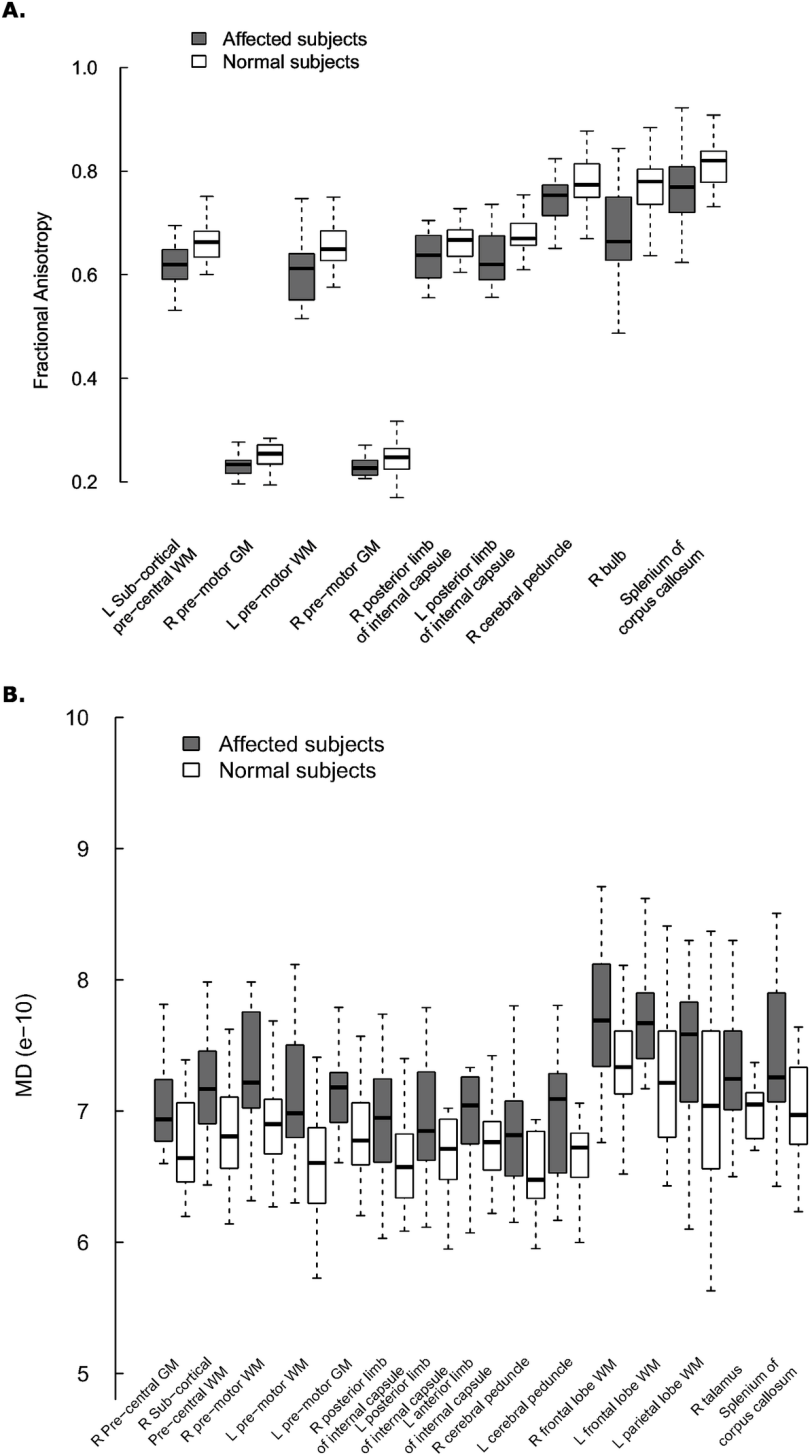
DTI values in controls and HSP patients for FA (A) and MD (B). Only anatomical areas with statistically significant differences are reported (ANOVA, *p-values* not adjusted for multiple comparisons ranged between 0.002 and 0.048). Abbreviations: of Fig 4: WM, White Matter; GM, Gray Matter; R, Right; L, Left.

Analyzing single anatomical area sampled with ROIs, on the basis of ANOVA test statistically significant differences between controls and HSP patients were detected for both FA and MD in scattered regions of the brain (Fig 4). In HSPs FA values were never higher and MD ones never lower than in controls.

**Fig 5:**
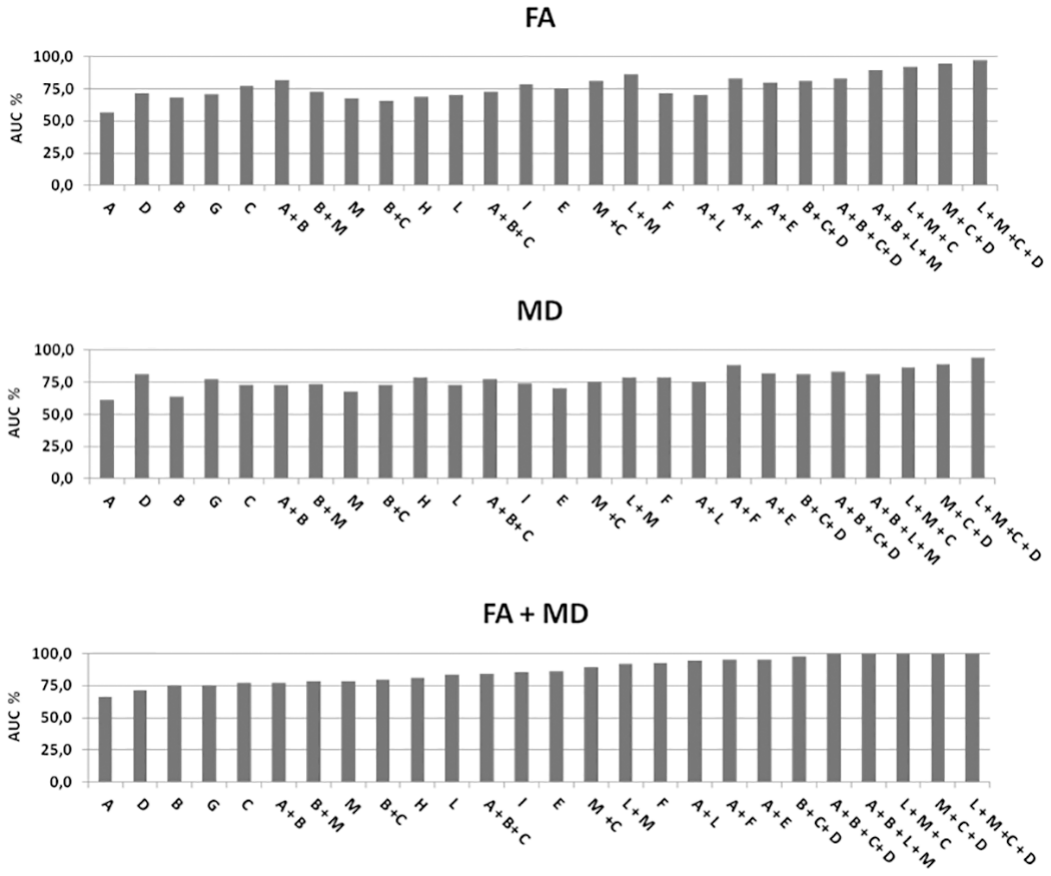
Discriminant analysis: Area under the ROC curve (AUC) expressed as percent (abscises axis), reported for fractional anisotropy (FA), mean diffusivity (MD) and FA+MD. Anatomical subdivision of cortico-spinal tract (CST) from pre-central and pre-motor grey matter (GM) to bulb: A, pre-central GM; B, sub-cortical pre-central white matter (WM); C, Deep supra-tentorial CST, from corona radiata to internal capsule; D, brain-stem CST tract, from cerebral peduncle to bulb; E, CST from sub-cortical pre-central WM to Cerebral Peduncles, throughout deep supra-tentorial WM; F, CST from sub-cortical pre-central WM to pons, throughout deep supra-tentorial WM; G, CST from corona radiata to cerebral peduncles; H, CST from corona radiata to pons; I, CST from corona radiata to bulb; L, Pre-motor GM; M, Pre-motor sub-cortical WM. X-axis: Combinations of the anatomical subdivision: A + B = pre-central GM and WM; B + C = Sub-cortical Pre-central WM and deep supra-tentorial CST; B + C + D = CST from sub-cortical pre-central WM to bulb, throughout deep supra-tentorial WM; A + B + C = Pre-central GM, more sub-cortical pre-central WM and deep supra-tentorial CST; A + E = Pre-central GM, more CST from sub-cortical pre-central WM to Cerebral Peduncles, throughout deep supra-tentorial; A + F = Pre-central GM, more CST from sub-cortical pre-central WM to pons, throughout deep supra-tentorial; A + B + C + D = Pre-central GM, more CST from sub-cortical pre-central WM to bulb, throughout deep supra-tentorial; L + M = Pre-motor GM and WM; A + L = Pre-central and pre-motor GM; B + M = Pre-central and pre-motor WM; A + B + L + M = Pre-central and pre-motor GM and WM; M + C = Pre-motor sub-cortical WM, more deep supra-tentorial CST, from corona radiata to internal capsule; L + M + C = Pre-motor GM, more Pre-motor sub-cortical WM and deep supra-tentorial CST, from corona radiata to internal capsule; M + C + D = Pre-motor sub-cortical WM, more deep CST, from corona radiata to bulb; L + M +C + D = Pre-motor GM and WM, more deep CST, from corona radiata to bulb.

Discriminant analysis was summarized as percentage of correct classification of patients (sensitivity) and healthy controls (specificity) and as area under the ROC curve (AUC) in segmentation of CST from the motor and pre-motor GM at the vertex, to the bulb, throughout the deep cerebral structures (Fig 5). MD never reached the 100% in sensitivity and specificity. Including the various portions of the CST, the percentage progressively increased from the vertex to the bottom (maximum 94.4% in specificity). Including the entire CST and the pre-motor GM, the percentages were higher than in aggregation with pre-central GM. Considering FA values, the 100% in specificity was already reached with the aggregation of the GM and the WM of the pre-central and pre-motor regions. Including the beneath tract of the CST, only when the pre-motor regions were added, the percentage of specificity was the highest. Considering the weight of both the DTI parameters (FA + MD), aggregation of WM and GM reached the 100% in specificity and sensitivity including the pre-central GM and the entire CST. When we considered pre-motor GM, the 100% in sensitivity/specificity was reached not only considering the entire CST, but already at the level of the internal capsule. The same 100% in sensitivity/specificity was detectable aggregating the GM of the pre-central and pre-motor regions and the beneath corresponding WM. GM of the pre-central and pre-motor regions individually never reached the 100% level of discriminating values, but the values were higher considering the pre-motor regions than the pre-central ones (sensitivity/specificity: pre-motor = 89.5%/94.4%; pre-central = 81.8%/72.7%).

Considering clinical and DTI data, SPRS scores correlated strongly with FA and MD for all the anatomical WM subdivision proposed (from *p*<0.05 to *p*<0.005).

## Discussion

We present the phenotype characterization of a large series of patients affected by HSP due to nine of the most frequent autosomal dominant and recessive SPG genotypes. The application of the multidisciplinary evaluation protocol allowed the clear identification of the commonalities shared by all forms and the peculiarities characterizing the individual genetic forms. HSP is a condition always affecting the long tracts with prevalent but not exclusive involvement of the longest upper motor neuron axons. The extension of the damage to the lower motor neuron and to the ascending tracts is recurrent albeit not uniform in the various molecular forms explored. The coupling of some predictive clinical scoring systems to the DTI findings and neurophysiology studies shows a promising and reliable way to define these forms with a detailed evidence of the motor system impairment as well as the involvement of other systems. Its power in monitoring disease progression and response to treatment needs to be tested in longitudinal studies, but the good correlation of both the DTI variables and the SPRS with disease duration seems a good promise.

The main weakness of this study is the unequal distribution of patients in the various SPG forms as well as the partially complete results from the paraclinical examinations that, given the low number of patients, further reduces the significance of the results. Variability of age and disease duration across the studied cohort and in the various SPG forms hinders comparability of disease specific subgroups. Some of these elements are typical of rare diseases, and therefore they are difficult to overcome. Nevertheless our cohort reflects the distribution of HSP genotypes described in most studies based in Western Europe [20, 43, 44]. One special point refers to children, representing a small share of the cohort, and for whom specially adapted clinical tools had to be used. The comparability of children specific and adult tools is only partially possible.

The first set of clinical variables is the chronological spectrum of SPG forms. In our cohort, the age of onset follows a bimodal distribution, with most juvenile forms (SPG 3a, 10, 11, 31) manifesting within the fifth year of life, and a second peak of the adult onset forms manifesting in the fourth and fifth decade. This distribution was already described in a SPG4 cohort [45], and thus it is not due to the locus specific characteristics of the different forms here considered. When we grouped the patients by genetic cause some characterizing elements of the specific forms could be identified. SPG4 is confirmed as the most frequent molecular form (45.7%), with higher prevalence in males [46] and age of onset usually in young adulthood. SPG3a is the form with the earliest age of onset, followed by SPG31. Adolescence to young adulthood is also the typical age at which symptoms are noted in SPG11, SPG15 and SPG5. The usual relative frequency of the most common dominant (SPG3a, SPG4, SPG10) and recessive (SPG 5, SPG 7, SPG 11) forms is confirmed by our cohort with the exception of, SPG31, reported as one of the most frequent early onset forms [9], but under-represented among our patients. In a recent study of a large cohort of HSP European patients all SPG31 detected (10%) came from France and none from Italy [47]. This may underline a strong dependence from the population genetic background for SPG31 [9, 48, 49], and should prompt appropriate comparison with other southern European HSP cohorts [17].

The second set of clinical variables is represented by the signs and symptoms pattern consistently detected in all SPG forms, and the measures linked to them. The LLs hyperactive DTRs and weakness are found in all subjects, but the degree of spasticity and involvement of lower motor neuron varies more across subjects than across SPG type. The uniform occurrence of significant weakness detected in our cohort was a surprise considering previous reports [2, 3]. Weakness is frequently overshadowed by the concurrent spasticity, but the systematic standardized application of muscle strength testing might be sufficient to uncover it.

Additional signs in HSPs are useful in discriminating the “pure” *vs* the “complicated” forms. Some additional features are certainly more common among the genetic forms labeled as “complicated” (SPG7, SPG11, SPG15, SPG35), but they are also met in other forms traditionally considered “pure” (SPG3a, SPG4).

Among the clinical and paraclinical indicators, we found that some of them, such as increased mCCT and DTI changes are useful in supporting the diagnosis while others, such as SPRS and possibly DTI are sensible tools for follow-up. SPRS reassumes most of the motor variables and also demonstrated a significant correlation with measures of autonomy, thus it represents an easy and reliable way to assess disease severity. The correlations of disease severity, as expressed by the SPRS, are stronger with disease duration than with age of onset. Thus age at onset per se is not predictive of disease severity, but at similar ages subjects with earlier onset will have a more severe phenotype than subjects with later onset.

The results of the neurophysiological and neuroimaging studies represent the third set of indicators. The neurophysiological assessment provided some confirmatory data and some novelties. A significant delay in LL mCCT was a constant feature in all but one patient, but the involvement of the motor and sensory tracts for the ULs found in a third of cases, even in absence of any clinical sign, is a clear indication that the condition is not exclusively affecting the longest tracts. Our results are in good agreement with other reports even though the reported cohorts are not fully comparable [20].

Routine morphological MRI examination has poor discriminating value for HSP [28, 50]. Even if the most part of our SPG11 patients showed a thin corpus callosum and the “ears of lynx” sign, WM alterations are very unspecific and not reflecting a specific involvement of the motor system [51]

On the contrary, our results confirm for DTI technique a very interesting novel role, shading light on the microstructural organization of the brain in HSP patients *in vivo*.

Comparing single ROI values grouped for anatomical region, FA and MD in HSP were statistically different in scattered areas involving both motor and extra-motor regions (Fig 4). This is not surprising, because other reports adopting DTI voxel based analysis demonstrated the widespread brain involvement in HSP [26, 52], both in the pure [24] and in complicated form [25].

Our analysis on DTI data is particularly interesting when we consider the discriminating capability of each CST tract to distinguish pathological versus normal pattern. The MD and FA single role are not completely exhaustive: the percentage of discriminating capability of MD values progressively increases including the various portions of the CST, never reaching 100%; the discriminating values are highest when to the whole CST is added the pre-motor GM than pre-central GM; FA values reached the 100% of specificity aggregating GM and WM of the pre-motor and pre-central regions or combining pre-motor GM and WM with the entire CST.

The discriminating power in distinguishing HSP patients from controls is very high when FA and MD are taken together. This confers to our DTI manipulation the character of a novel approach in discriminating patients from controls: only a short tract of the CST is needed for the identification of patients, especially when the pre-motor regions are sampled. The involvement of pre-motor area in other MND was already reported in other DTI studies [53] and its role is very intriguing in our cohort of patient.

The predictive value of the indicators described in this paper can be established only through a prospective study, which is currently underway. Indeed, our expectation is that SPRS and DTI may serve as indicators of disease progression and could signal response to treatment. A recent report about DTI application on SPG4 genotype underlies the opportunity of applying DTI methods to evaluate disease duration and severity [27].

The predictive value of the indicators described in this paper can be established only through a prospective study, which is currently underway. Indeed, our expectation is that SPRS and DTI may serve as indicators of disease progression and could signal response to treatment.

Recent studies point towards a growing connection among the pathophysiological mechanisms underlying the various genetic forms of HSPs [19, 28] suggesting, at least from a certain point of the pathogenic cascade on, a common pathway responsible for the observed axonal damage of the long tracts [27, 54, 55].

For this reason it seems appropriate to consider, as we did in this study, the composite and heterogeneous group of HSPs as a metasyndromic umbrella sharing a common way to produce and manifest pathology. The bewildering (and still growing) variety of possible genes involved in causing HSP and the lack of reliable and specific biomarkers of disease are challenges clinicians have to face when trying to efficiently reach a definite diagnosis, provide a sensible way to monitor disease progression and offer a reliable prognosis. In this respect the recommendations that emerge from our study for disease diagnosis & characterization are summarized in Table 4. In this table also potential tools for disease progression and severity are shown.

**Table 4:** Recommendations on the assessment tools for clinical use in HSP patients.

Assessment/indicator	Tools for disease diagnosis & characterization	Tools for disease progression and severity
**SPRS / score**		X
**MMT / MRC megascore**	X	
**DTR / mean score**	X	
**6MWT /mt**	X	
**MEP / mCTT**	X	
**MRI / FA & MD of CST**	X	Possibly

**Abbreviations**: SPRS, spastic paraplegia rating scale; MMT, manual muscle testing; MRC, Medical Research Council; DTR, deep tendon reflexes; 6mWT, 6 minute walking test; MEP, motor evoked potentials; mCCT, motor central conduction time; MRI, magnetic resonance imaging; FA, fractional anisotropy; MD, mean diffusivity; CST, cortico-spinal tract.

In conclusion, by looking in a uniform way at a large group of heterogeneous HSP patients we have shown for the first time a common quantitative alteration in MRI parameters which correlates with clinical variables. We gathered further indications that the phenotype in HSP is exceeding the motor system alone. Our findings will now need to be replicated, possibly with longitudinal extension, in other large independent cohorts.

## Supporting Information

S1 File This section contains the following material. Supplementary findings: Genetic Profile of the patients, ROIs delineation for DTI sampling, H-MR spectroscopy results; S1 Supplementary References.
(PDF)

S1 Table Mutation detected in the reported patients. (PDF)

S2 Table Clinical-functional data of the patients studied. (PDF)

## Abbreviations

Cr: creatine-phosphocreatine complex
CST: cortico-spinal tract
DTI: diffusion tensor imaging
DTR: deep tendon reflexes
ENB2: brief neuropsychological inventory 2
EP: evoked potentials
FA: fractional anisotropy
GM: grey matter
ICC: intra-class correlation coefficient
FIM: functional independence measure
HSP: hereditary spastic paraplegia
IQ: intellectual quotient
LL: lower limb
MAS: modified Ashworth scale
MCA: multiple correspondence analysis
mCCT: motor central conduction time
MD: mean diffusivity
MER: motor evoked responses
MND: motor neuron disease
MRC: Medical Research Council
MRS: magnetic resonance spectroscopy
NCS: nerve conduction studies
ROI: region of interest
SC: standardized coefficient
SD: standard deviation
SPRS: spastic paraplegia rating scale
SSEP: somatosensory evoked potentials
UL: upper limb
WM: white matter
6mWT: 6 minute walking test
