# Supplementary material for: Clinical and Paraclinical Indicators of Motor System Impairment in Hereditary Spastic Paraplegia: A Pilot Study
Source: PLoS One. 2016 Apr 14;11(4):e0153283. doi: 10.1371/journal.pone.0153283 (PMC4831837; doi:10.1371/journal.pone.0153283)
Supplement: S1 File — Supplementary findings: Genetic Profile of the patients, ROIs delineation for DTI sampling, H-MR spectroscopy results; S1 Supplementary References. (PDF) [file pone.0153283.s001.pdf]

Supporting information S1

Table of contents

1. Supplementary findings: Genetic Profile of the patients
2. ROIs delineation for DTI sampling
3. H-MR spectroscopy results
4. S1 Supplementary References

1. Supplementary findings: Genetic Profile of the patients

From the genetic testing, 19 patients were found to have new mutation. Three 3 new deletions in SPG4: c.1620_1623delGACT/InsC (3 related patients), exon 16 deletion (MLPA) (1 patient), exon 17 deletion (MLPA) (4 patients) and one deletion in SPG11 c.408_428del21 p.E136_I142del (1 patients). Other 10 mutations were found in 10 SPG4 patients (S1 Table).

2. ROIs delineation for DTI sampling

All delineations of the Regions of Interest (ROIs) were performed by three operators (26, 16 and 3 years of experience, respectively for DM, HH and MV). The ROIs were applied to the DTI maps in 2 different stages: an instruction stage, during which in two weeks of daily work, the accuracy of MRI anatomical location of the ROIs were discussed among the three operators and then reconstructed for all the 22 normal subjects and repeated for the patient group. When the accordance of anatomical positioning and the corresponding FA and MD values were close to both the operator, in the last stage two operators (HH and MV) independently created individual data set, extracting single FA and MD values for each volunteer and patient; inter-observer agreement was also calculated (HH *vs* MV). ROIs positioned by the second operator (MV) were repeated twice in order to study intra-observer agreement.

3. H-MR spectroscopy results

By H-MRS samplings no significant differences between HPS patients and controls were identified. The recent reports on MRS technique concerning some minimal variations in HSP patients [S25-S27] are not confirmed in this study: we found no differences in 1H-MRS metabolic profile between HSP patients and controls and no correlation with clinical/paraclinical variables. Possible explanations could be the limited strength of the magnet (1.5T), the limited dimension of the sampling volume and the nature of the possible metabolic variation itself, which may be too subtle to be detected in a limited number of patients.

4. S1 Supplementary References

S1 Supplementary References

S1. Patrono C, Scarano V, Cricchi F, Melone MA, Chiriaco M, Napolitano A, Malandrini A, De Michele G, Petrozzi L, Giraldi C, Santoro L, Servidei S, Casali C, Filla A, Santorelli FM. Autosomal dominant hereditary spastic paraplegia: DHPLC-based mutation analysis of SPG4 reveals eleven novel mutations. Hum Mutat. 2005 May;25(5):506. PMID: 15841487.

S2. Fonknechten N, Mavel D, Byrne P, Davoine CS, Cruaud C, Bönsch D, Samson D, Coutinho P, Hutchinson M, McMonagle P, Burgunder JM, Tartaglione A, Heinzlef O, Feki I, Deufel T, Parfrey N, Brice A, Fontaine B, Prud'homme JF, Weissenbach J, Dürr A, Hazan J. Spectrum of SPG4 mutations in autosomal dominant spastic paraplegia. Hum Mol Genet. 2000 Mar 1;9(4):637-44.

S3. Crippa F, Panzeri C, Martinuzzi A, Arnoldi A, Redaelli F, Tonelli A, Baschirotto C, Vazza G, Mostacciuolo ML, Daga A, Orso G, Profice P, Trabacca A, D'Angelo MG, Comi GP, Galbiati S, Lamperti C, Bonato S, Pandolfo M, Meola G, Musumeci O, Toscano A, Trevisan CP, Bresolin N, Bassi MT. Eight novel mutations in SPG4 in a large sample of patients with hereditary spastic paraplegia. Arch Neurol. 2006 May;63(5):750-5.

S4. Depienne C, Tallaksen C, Lephay JY, Bricka B, Poea-Guyon S, Fontaine B, Labauge P, Brice A, Durr A. Spastin mutations are frequent in sporadic spastic paraparesis and their spectrum is different from that observed in familial cases. J Med Genet. 2006 Mar;43(3):259-65. Epub 2005 Jul 31.

S5. Meijer IA, Hand CK, Cossette P, Figlewicz DA, Rouleau GA. Spectrum of SPG4 mutations in a large collection of North American families with hereditary spastic paraplegia. Arch Neurol. 2002 Feb;59(2):281-6.

S6. Magariello A, Muglia M, Patitucci A, Mazzei R, Conforti FL, Gabriele AL, Sprovieri T, Ungaro C, Gambardella A, Mancuso M, Siciliano G, Branca D, Aguglia U, de Angelis MV, Longo K, Quattrone A. Novel spastin (SPG4) mutations in Italian patients with hereditary spastic paraplegia. Neuromuscul Disord. 2006 Jun;16(6):387-90. Epub 2006 May 8.

S7. Blair MA, Riddle ME, Wells JF, Breviu BA, Hedera P. Infantile onset of hereditary spastic paraplegia poorly predicts the genotype. Pediatr Neurol. 2007 Jun;36(6):382-6.

S8. Park SY, Ki CS, Kim HJ, Kim JW, Sung DH, Kim BJ, Lee WY. Mutation analysis of SPG4 and SPG3A genes and its implication in molecular diagnosis of Korean patients with hereditary spastic paraplegia. Arch Neurol. 2005 Jul;62(7):1118-21.

S9. Hentati A, Deng HX, Zhai H, Chen W, Yang Y, Hung WY, Azim AC, Bohlega S, Tandan R, Warner C, Laing NG, Cambi F, Mitsumoto H, Roos RP, Boustany RM, Ben Hamida M, Hentati F, Siddique T. Novel mutations in spastin gene and absence of correlation with age at onset of symptoms. Neurology. 2000 Nov 14;55(9):1388-90.

S10. Dalpozzo F, Rossetto MG, Boaretto F, Sartori E, Mostacciuolo ML, Daga A, Bassi MT, Martinuzzi A. Infancy onset hereditary spastic paraplegia associated with a novel atlastin mutation. Neurology. 2003 Aug 26;61(4):580-1.

S11. Sauter SM, Engel W, Neumann LM, Kunze J, Neesen J. Novel mutations in the Atlastin gene (SPG3A) in families with autosomal dominant hereditary spastic paraplegia and evidence for late onset forms of HSP linked to the SPG3A locus. Hum Mutat. 2004 Jan;23(1):98. PMID: 14695538.

S12. Goizet C, Boukhris A, Durr A, Beetz C, Truchetto J, Tesson C, Tsaousidou M, Forlani S, Guyant-Maréchal L, Fontaine B, Guimarães J, Isidor B, Chazouillères O, Wendum D, Grid D, Chevy F, Chinnery PF, Coutinho P, Azulay JP, Feki I, Mochel F, Wolf C, Mhiri C, Crosby A, Brice A, Stevanin G. CYP7B1 mutations in pure and complex forms of hereditary spastic paraplegia type 5. Brain. 2009 Jun;132(Pt 6):1589-600. doi: 10.1093/brain/awp073. Epub 2009 May 12.

S13. Arnoldi A, Crimella C, Tenderini E, Martinuzzi A, D'Angelo MG, Musumeci O, Toscano A, Scarlato M, Fantin M, Bresolin N, Bassi MT. Clinical phenotype variability in patients with hereditary spastic paraplegia type 5 associated with CYP7B1 mutations. Clin Genet. 2012 Feb;81(2):150-7. doi: 10.1111/j.1399-0004.2011.01624.x. Epub 2011 Jan 31.

S14. McDermott CJ, Dayaratne RK, Tomkins J, Lusher ME, Lindsey JC, Johnson MA, Casari G, Turnbull DM, Bushby K, Shaw PJ. Paraplegin gene analysis in hereditary spastic paraparesis (HSP) pedigrees in northeast England. Neurology. 2001 Feb 27;56(4):467-71.

S15. Arnoldi A, Tonelli A, Crippa F, Villani G, Pacelli C, Sironi M, Pozzoli U, D'Angelo MG, Meola G, Martinuzzi A, Crimella C, Redaelli F, Panzeri C, Renieri A, Comi GP, Turconi AC, Bresolin N, Bassi MT. A clinical, genetic, and biochemical characterization of SPG7

mutations in a large cohort of patients with hereditary spastic paraplegia. Hum Mutat. 2008 Apr;29(4):522-31. doi: 10.1002/humu.20682.

S16. Crimella C1, Baschirotto C, Arnoldi A, Tonelli A, Tenderini E, Airoldi G, Martinuzzi A, Trabacca A, Losito L, Scarlato M, Benedetti S, Scarpini E, Spinicci G, Bresolin N, Bassi MT. Mutations in the motor and stalk domains of KIF5A in spastic paraplegia type 10 and in axonal Charcot-Marie-Tooth type 2. Clin Genet. 2012 Aug;82(2):157-64. doi: 10.1111/j.1399-0004.2011.01717.x. Epub 2011 Jun 21.

S17. Musumeci O, Bassi MT, Mazzeo A, Grandis M, Crimella C, Martinuzzi A, Toscano A. A novel mutation in KIF5A gene causing hereditary spastic paraplegia with axonal neuropathy. Neurol Sci. 2011 Aug;32(4):665-8. doi: 10.1007/s10072-010-0445-8. Epub 2010 Nov 24.

S18. Stevanin G, Santorelli FM, Azzedine H, Coutinho P, Chomilier J, Denora PS, Martin E, Ouvrard-Hernandez AM, Tessa A, Bouslam N, Lossos A, Charles P, Loureiro JL, Elleuch N, Confavreux C, Cruz VT, Ruberg M, Leguern E, Grid D, Tazir M, Fontaine B, Filla A, Bertini E, Durr A, Brice A. Mutations in SPG11, encoding spatacsin, are a major cause of spastic paraplegia with thin corpus callosum. Nat Genet. 2007 Mar;39(3):366-72. Epub 2007 Feb 18.

S19. Crimella C, Arnoldi A, Crippa F, Mostacciuolo ML, Boaretto F, Sironi M, D'Angelo MG, Manzoni S, Piccinini L, Turconi AC, Toscano A, Musumeci O, Benedetti S, Fazio R, Bresolin N, Daga A, Martinuzzi A, Bassi MT. Point mutations and a large intragenic

deletion in SPG11 in complicated spastic paraplegia without thin corpus callosum. J Med Genet. 2009 May;46(5):345-51. doi: 10.1136/jmg.2008.063321. Epub 2009 Feb 5.

S20. Hehr U, Bauer P, Winner B, Schule R, Olmez A, Koehler W, Uyanik G, Engel A, Lenz D, Seibel A, Hehr A, Ploetz S, Gamez J, Rolfs A, Weis J, Ringer TM, Bonin M, Schuierer G, Marienhagen J, Bogdahn U, Weber BH, Topaloglu H, Schols L, Riess O, Winkler J. Long-term course and mutational spectrum of spatacsin-linked spastic paraplegia. Ann Neurol. 2007 Dec;62(6):656-65.

S21. Stevanin G, Azzedine H, Denora P, Boukhris A, Tazir M, Lossos A, Rosa AL, Lerer I, Hamri A, Alegria P, Loureiro J, Tada M, Hannequin D, Anheim M, Goizet C, Gonzalez-Martinez V, Le Ber I, Forlani S, Iwabuchi K, Meiner V, Uyanik G, Erichsen AK, Feki I, Pasquier F, Belarbi S, Cruz VT, Depienne C, Truchetto J, Garrigues G, Tallaksen C, Tranchant C, Nishizawa M, Vale J, Coutinho P, Santorelli FM, Mhiri C, Brice A, Durr A; SPATAX consortium. Mutations in SPG11 are frequent in autosomal recessive spastic paraplegia with thin corpus callosum, cognitive decline and lower motor neuron degeneration. Brain. 2008 Mar;131(Pt 3):772-84. Epub 2007 Dec 13.

S22. Vantaggiato C, Crimella C, Airoldi G, Polishchuk R, Bonato S, Brighina E, Scarlato M, Musumeci O, Toscano A, Martinuzzi A, Santorelli FM, Ballabio A, Bresolin N, Clementi E, Bassi MT. Defective autophagy in spastizin mutated patients with hereditary spastic paraparesis type 15. Brain. 2013 Oct;136(Pt 10):3119-39. doi: 10.1093/brain/awt227. Epub 2013 Sep 11.

S23. Battini R, Fogli A, Borghetti D, Michelucci A, Perazza S, Baldinotti F, Conidi ME, Ferreri MI, Simi P, Cioni G. Clinical and genetic findings in a series of Italian children with pure hereditary spastic paraplegia. Eur J Neurol. 2011 Jan;18(1):150-7. doi: 10.1111/j.1468-1331.2010.03102.x.

S24. Tonelli A, D'Angelo MG, Arrigoni F, Brighina E, Arnoldi A, Citterio A, Bresolin N, Bassi MT. Atypical adult onset complicated spastic paraparesis with thin corpus callosum in two patients carrying a novel FA2H mutation. Eur J Neurol. 2012 Nov;19(11):e127-9. doi: 10.1111/j.1468-1331.2012.03838.x. Epub 2012 Aug 27.

S25. Erichsen AK, Server A, Landro NI, Sandvik L, Tallaksen CM. Proton magnetic resonance spectroscopy and cognition in patients with spastin mutations. J Neurol Sci 2009; 277: 124-9.

S26. Roos P, Svenstrup K, Danielsen ER, Thomsen C, Nielsen JE. CYP7B1: novel mutations and magnetic resonance spectroscopy abnormalities in hereditary spastic paraplegia type 5A. Acta Neurol Scand 2014; 129: 330-4.

S27. Stromillo ML, Malandrini A, Dotti MT, Battaglini M, Borgogni F, Tessa A, et al. Structural and metabolic damage in brains of patients with SPG11-related spastic paraplegia as detected by quantitative MRI. J Neurol 2011; 258: 2240-7.
