# Supplementary material for: Clinical and Paraclinical Indicators of Motor System Impairment in Hereditary Spastic Paraplegia: A Pilot Study
Source: PLoS One. 2016 Apr 14;11(4):e0153283. doi: 10.1371/journal.pone.0153283 (PMC4831837; doi:10.1371/journal.pone.0153283)
Supplement: S1 Table — (PDF) [file pone.0153283.s002.pdf]

**S1 Table Mutation detected in the reported patients**

| Patient's code | MUTATION | Type | GENE | Reference |
|---|---|---|---|---|
| **#1** | **c.1413+1G>A** | **Splicing** | **SPG4** | [S1] |
| **#2** | **c.1620_1623delGACT/InsC** | **Deletion** | **SPG4** | |
| **#3** | **c.1620_1623delGACT/InsC** | **Deletion** | **SPG4** | |
| **#4** | **exon 17 deletion (MLPA)** | **Deletion** | **SPG4** | |
| **#5** | **exon 17 deletion (MLPA)** | **Deletion** | **SPG4** | |
| **#6** | **c.1709A>G p.K570R** | **Missense** | **SPG4** | |
| **#7** | **c.1763C>A p.S588Y** | **Missense** | **SPG4** | |
| **#8** | **c.1634G>C p.S545X** | **Truncating** | **SPG4** | |
| **#9** | **c.1291C>T p.R431X** | **Truncating** | **SPG4** | [S2] |
| **#10** | **c.1222A>G, p.M408V** | **Missense** | **SPG3a** | [S10] |
| **#11** | **c.1222A>G, p.M408V** | **Missense** | **SPG3a** | [S10] |
| **#12** | **c.1222A>G, p.M408V** | **Missense** | **SPG3a** | [S10] |
| **#13** | **c.1222A>G, p.M408V** | **Missense** | **SPG3a** | [S10] |
| **#14** | **c.889A>G p.Y297A exon 4 / c.260G>T p.G87V** | **Missense** | **SPG5** | [S12] |
| **#15** | **c.889A>G p.Y297A exon 4 / c.260G>T p.G87V** | **Missense** | **SPG5** | [S12] |
| **#16** | **c.605G>A; p.S202N** | **Missense** | **SPG10** | [S16] |
| **#17** | **exon 2-5 Deletion (MLPA)** | **Deletion** | **SPG31** | [S23] |
| **#18** | **c.733_734delAT/p.M245VfsX / c.7081insT p.F2361FfsX** | **truncating** | **SPG11** | [S18] |
| **#19** | **c.1450G>C p.G484R** | **Missense** | **SPG4** | |
| **#20** | **exon 16 deletion (MLPA)** | **Deletion** | **SPG4** | |
| **#21** | **c.1456C>T p.R486C** | **Missense** | **SPG5** | [S12] |
| **#22** | **c.1456C>T p.R486C** | **Missense** | **SPG5** | [S12] |
| #23 | c.1620_1623delGACT/InsC | Deletion | SPG4 | |
| #24 | exon 17 deletion (MLPA) | Deletion | SPG4 | |
| #25 | exon 17 deletion (MLPA) | Deletion | SPG4 | |
| #26 | c.1291C>T p.R431X | Truncating | SPG4 | [S2] |

| | | | | |
|---|---|---|---|---|
| #27 | c.1222A>G, p.M408V | Missense | SPG3a | [S10] |
| #28 | c.583C>G p.L195V | Missense | SPG4 | [S3] |
| #29 | c.1507C>T p.R503W | Missense | SPG4 | [S4] |
| #30 | c.1092G>T, p.R364S | Missense | SPG4 | |
| #31 | c.439G>T p.E147X | Truncating | SPG4 | [S1] |
| #32 | c.439G>T p.E147X | Truncating | SPG4 | |
| #33 | c.583C>G p.L195V | Missense | SPG4 | [S3] |
| #34 | c.1246-1G>T IVS9 | Missense | SPG4 | |
| #35 | c.1821G>C p.W607C | Missense | SPG4 | [S3] |
| #36 | c.1322 -2 A>C | Splicing | SPG4 | |
| #37 | c.1685G>A p.R562Q | Missense | SPG4 | [S5] |
| #38 | c.439G>T p.E147X | Truncating | SPG4 | [S1] |
| #39 | c.326C>G p.P109R | Missense | SPG4 | [S6] |
| #40 | c.1537G>A p.G471D | Missense | SPG4 | [S7] |
| #41 | c.854A>T p.H285L | Missense | SPG5 | [S13] |
| #42 | c.854A>T p.H285L | Missense | SPG5 | [S13] |
| #43 | c.679C>T , p.R227X //c.1231G>A p.D412N | Truncating/Missense | SPG7 | [S14] |
| #44 | c.609C>G, p.S203C | Missense | SPG10 | [S17] |
| #45 | c.609C>G, p.S203C | Missense | SPG10 | [S17] |
| #46 | c.5014G>T E1672X / c.6100C>T R2034X | Truncating | SPG11 | [S18] |
| #47 | c.733-734delAT/p.M245VfsX / c.6157G>A p.V2053M | Truncating/Missense | SPG11 | [S18; S19] |
| #48 | c.2833A>G p.R945GfsX950 | Truncating | SPG11 | [S18] |
| #49 | c.3935C>A p.S1312X | Truncating | SPG15 | [S22] |
| #50 | c.1550G>T p.L517W | Missense | SPG4 | |

| | | | | |
|---|---|---|---|---|
| #51 | c.404A>G p.E135G | Missense | SPG4 | |
| #52 | c.1496G>A p.R499H | Missense | SPG4 | [S8] |
| #53 | c.334G>T p.E112X | Truncating | SPG4 | [S9] |
| #54 | c.715C>T, p.R239C | Missense | SPG3a | [S11] |
| #55 | c.650G>A, p.R217Q; | Missense | SPG3a | [S11] |
| #56 | c.1328G>C p.G443A | Missense | SPG5 | [S13] |
| #57 | c.1529C>T, p.A510V | Missense | SPG7 | [S14] |
| #58 | c.1529C>T, p.A510V | Missense | SPG7 | [S14] |
| #59 | c.233T>A p.Leu78X | Truncating | SPG7 | [S15] |
| #60 | c.408_428del21 p.E136_I142del | Deletion | SPG11 | |
| #61 | c.349G>T p.E117X / c.2833A>G p.R945G | Truncating/Missense | SPG11 | [S18; S19] |
| #62 | c.442+1G>C IVS2 / c.1735+2_7del AAGT IVS8 | Splicing | SPG11 | [S20] |
| #63 | c. 5986_5987insT p.C1996LfsX1999 / c.6418C>T Q2140X | Truncating | SPG11 | [S21] |
| #64 | c.1203delA p.K401KfsX | Truncating | SPG11 | [S18] |
| #65 | c.442+1G>C IVS2 / c.4161 -1G>A | Splicing | SPG11 | [S20] |
| #66 | c.6832_6833delAG p.S2278LfsX2338 | Truncating | SPG11 | [S21] |
| #67 | c.1523T>A p.I508N | Missense | SPG15 | [S22] |
| #68 | c.1523T>A p.I508N | Missense | SPG15 | [S22] |
| #69 | c.509A>G p.Y170C | Missense | SPG35 | [S24] |
| #70 | c.509A>G p.Y170C | Missense | SPG35 | [S24] |

**Note:** In bold patients that underwent the DTI and MRS protocol (n=22). References are found in the final part of the present document.
**Abbreviations:** MLPA = Multiplex Ligation-dependent Probe Amplification, SPG = Spastic Paraplegia Gene.
