# Supplementary material for: Clinical and Paraclinical Indicators of Motor System Impairment in Hereditary Spastic Paraplegia: A Pilot Study
Source: PLoS One. 2016 Apr 14;11(4):e0153283. doi: 10.1371/journal.pone.0153283 (PMC4831837; doi:10.1371/journal.pone.0153283)
Supplement: S2 Table — (PDF) [file pone.0153283.s003.pdf]

S2 Table. Clinical-functional data of the patients studied

Parameters	n	mean ± SD (range)	Normal values
SPRS	69	23.27 ± 10.74 (4-48)	0
6mWT (mt)	40	220.82 ± 104.37 (64.8-412.2)	615,5 ± 50,5 [39]
FIM	37	108.32 ± 20.76 (55-126)	126
Muscle tone	70	2.04 ± 0.98 (0-4)	0
Muscle strength	70	27.23 ± 12.3 (0-40)	40
DTR	70	2.95 ± 0.88 (0-4)	2

Abbreviations: 6MWT = (mt) 6-Minute Walking Test, normal values are mean between women and men mean values as reported in Chetta et al. [41]; DTR = Deep Tendon Reflexes (range 0-4); FIM = Functional Independence Measure (range 18-126); Muscle strength = MRC medical research council (range for single or muscle group 0-5, range for the MRC megascore 0-40); Muscle tone = Modified Ashworth Scale (range 0-4); SPRS = Spastic Paraplegia Rating Scale (range 0-52).
